# Boundarics in Biomedicine

**DOI:** 10.34133/research.0430

**Published:** 2024-08-09

**Authors:** Quansheng Du, Juan Li, Fang Yang, Hui Dai, Aiguo Wu

**Affiliations:** ^1^ National Natural Science Foundation of China, Beijing 100085, China.; ^2^ Laboratory of Advanced Theranostic Materials and Technology, Ningbo Institute of Materials Technology and Engineering, Chinese Academy of Sciences, Ningbo 315201, China.

## Abstract

“Boundarics in Biomedicine” is a cutting-edge interdisciplinary discipline, which is of great significance for understanding the origin of life, the interaction between internal and external environments, and the mechanism of disease occurrence and evolution. Here, the definition of Boundarics in Biomedicine is first described, including its connotation, research object, research method, challenges, and future perspectives.

“Boundarics in Biomedicine” is a cutting-edge interdisciplinary discipline involving multiple fields (e.g., bioscience, medicine, chemistry, materials science, and information science) dedicated to investigating and solving key scientific questions in the formation, identification, and evolution of living organism boundaries. Specifically, it encompasses 3 levels: (a) the boundary between the living organism and the external environment, (b) internal boundary within living organism, and (c) the boundary related to disease in living organism. The advancement of research in Boundarics in Biomedicine is of great scientific significance for understanding the origin of life, the interaction between internal and external environments, and the mechanism of disease occurrence and evolution, thus providing novel principles, technologies, and methods for early diagnosis and prevention of major diseases, personalized drug development, and prognosis assessment (Fig. 1).

## Boundary between the Living Organism and External Environment

From the perspective of life science, one of the defining attributes between life and non-life is the boundary. Physicist Erwin Schrödinger proposed in his work “What is Life?” that living complex system of life is a “trinity” of matter, energy, and information, with the most important characteristic being the emergence of life functions [1]. This emergence arises at boundary stratification and when realizing leaps, while hardly or not at the same level. Therefore, new life phenomena, regular mechanisms, and life construction rules generally exist at different levels and interfaces in living organisms. For instance, from the evolution of microorganisms, plants, and animals to humans, the boundaries are clearly delineated, which is also evident in the differentiation between life and non-life [2].

**Fig. 1.**
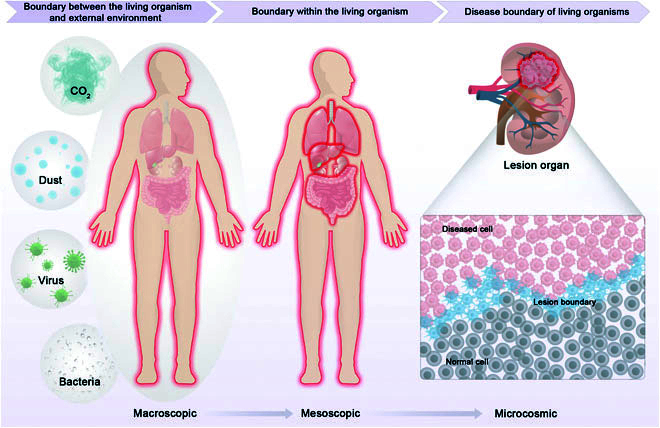
Schematic illustration of Boundarics in Biomedicine.

In physics, life is a dissipative structure and an open system far from thermal equilibrium. Due to the dissipative structure characteristics, life must constantly acquire matter and energy from its surroundings to maintain its self-stabilizing state, thus creating a boundary between life and its external environment [3]. Without this boundary to maintain a self-stabilizing state, life and its surroundings would merge, and the birth of life would be impossible. Therefore, the boundary is crucial to life’s origin and evolution.

From the perspective of informatics, life is a scale-free network with numerous nodes, which is different from known big data models constrained by algorithms [4]. However, biological data are usually reticulated, exhibiting correlation and association strength rather than causation. For example, the perception of yeast involves its genes, transcripts, proteins, etc., which are analyzed based on their correlations [5]. In addition, a few key nodes control other nodes in this network, regulating the entire living system through the massive reuse of simple rules, such as the central law or information flow rule. In this sense, the scale-free network drives the formation of biological boundaries and specific species.

Typical boundary issues exist among living organisms, e.g., the boundaries between life evolution, material world, and living beings. According to the current mainstream research logic, from individual tissues, cells, and molecules to atoms, the elemental composition of life is interconnected with the material world, without essential difference. However, numerous scientific challenges remain, such as how life can arise, where the demarcation point is, and how life constitutes a self-stabilizing system with its own distinctive features (e.g., genetics and metabolism).

Boundaries between living organisms and their environment are ubiquitous. No form of life can exist apart from the environment, and all life must be environmentally adapted. Simultaneously, the environment plays a significant role in molding the life morphology. For example, genetic isolation exists in the evolution of living organisms, and morphologically large differences may be evolutionarily derived from close branches [6]. Especially in the recent situation of relatively high climate fluctuations and elevated carbon dioxide levels, boundary investigations that help life adapt to rapidly altering world and climate conditions, support sustainable development, provide sufficient energy and a healthy living environment, and safeguard human health have been a hot and enduring topic [7].

## Boundary within the Living Organism

Natural boundaries exist within living organisms to maintain their structural functions and homeostatic balance, including the boundaries among the systems, organs, tissues, cells, and molecules. These boundaries play vital physiological functions in organisms for survival and health, as physical isolation layers and functional regulatory interfaces.

The 8 major systems that make up the human body are relatively independent, with significant boundaries between each other. Meanwhile, these systems coordinate to support the system’s integrity and ensure the normal progression of various complex life activities. In addition, the study of the boundaries of tissues and organs (e.g., heart, liver, spleen, lungs, kidneys, and brain) involves their separation, integration, and interaction [8]. Thereinto, the blood–brain barrier (BBB) is a highly functionalized central nervous system border that regulates substance transport between the blood and brain [9]. Even at a smaller level, the cell boundaries mainly focus on their cell membrane structure and functions. The cell membrane serves as a physical boundary, separating the cell from the extracellular environment, leading to multiple roles of protecting the cell against external substances, regulating the substance exchange, and enabling intercellular communication [10]. Additionally, the spatiotemporal evolutionary processes of significant molecules (e.g., proteins, nucleic acids, peptides, and small molecules) and their molecular events during boundary formation are essential for disease onset, early diagnosis, and developmental monitoring.

Currently, the boundary discernment of living organisms consists of visual observation, detection based on advanced biomedical probes, technologies, equipment, and means that are not yet verifiable by humans [11–13]. For example, the opening of Qi in traditional Chinese medicine extends beyond the structurally visible boundaries. As boundaries in physiological anatomy are generally static, there is a need to further explore and reflect on the dynamically changing and spatiotemporally evolving boundaries.

## Disease Boundary of Living Organisms

In general, there are homeostatic boundaries between different units in a living organism that can maintain normal biological functions. These boundaries are energetically stable, dynamically balanced, and evolutionarily changing with the 4-dimensional spatiotemporal domain. Once external influences disrupt the homeostasis between units and evolve with the spatiotemporal domain, their normal physiological functions are altered, extending to the molecules, cells, tissues, organs, living organisms, and other organismic units throughout the living system, thus resulting in pathological features [14]. Therefore, boundary anomalies are closely related to life health, and the boundaries formed by diseases in diverse occurrence, development, and evolution processes are essential for their early detection and diagnosis, which is the key to precise treatment and prognosis.

Theoretically, a cell is different from its normal neighboring cells in the early change stages, forming a hardly distinguishable boundary. As it further gradually affects the surrounding cell population, there is detectable structure formation. Therefore, detecting boundaries between normal and abnormal states in molecules, cells, or tissues at a remarkably early stage is significant for early disease detection, diagnosis, and treatment [15,16]. A relevant example in this field is the work of Zhou’s group [14], who detected a high degree of cellular and transcriptional heterogeneities in a 500-μm-wide invasive zone, thereby elucidating the mechanisms of tumor infiltration and invasion around the border. For early disease detection and diagnosis within the boundary region, Pal et al. [17] demonstrated that the fluorescence lifetime of tumor tissue is longer than the fluorescence lifetime of noncancerous tissue after indocyanine green injection. In addition, Tian and coworkers [13] described an optical-imaging instrument integrating a visible, multispectral imaging system to detect near infrared II (NIR-II) and NIR-I fluorescence for the fluorescence-guided surgical resection of primary and metastatic liver tumors in 23 patients. Nevertheless, the current technological tools for boundary identification, including biomedical imaging and molecular probe labeling, need to be revised to meet the demand for in-depth biomedical boundary research [18–20]. With increasing investment and development, new imaging or detection technologies, tools, and methods will emerge in the future. For instance, developing electronic biomedical technologies is a potentially effective way of solving problems in the border area, which is beneficial for efficient and precise medical diagnosis, treatment, and monitoring under dynamic transformation conditions [21–23].

Regarding lesion boundaries, the critical event molecules and their interactions within the boundary region, and the impact of such interactions on disease’s onset and progression, are of great interest. Parallel to this, it is significant to intervene or prevent the disease onset and progression through practical means and methods, as well as to reverse the developmental process as an efficient treatment [24–26]. Nano-transportation robots that can manipulate substances on a microscopic scale and advanced implantable drug delivery systems with high drug loading capacity, low side effects, and high bioavailability provide these technological approaches with unique advantages and research and development potential for addressing molecular events at local boundaries [27–30].

In addition, the nervous system can generate conditioned stimulus reflexes and achieve high-level activities. There are also boundaries between human language, art, consciousness, culture, matter, and spirit. The interfacial interplay between the nervous system and high-level creation is also worthy of eternal exploration.

## Challenges and Future Trends in Boundarics in Biomedicine

During the 21st century, the concept and recognition of Boundarics in Biomedicine have received considerable attention from researchers and scholars around the world. However, numerous major scientific and technical issues remain to be further investigated, e.g., the dynamics and complexity of cell boundaries, the boundary specificity between lesions and normal tissues and organs, and the interaction complexity between the internal and external environments of different organisms. Besides, the limitations of existing biomedical imaging technologies and the inadequacies of computational modeling theories also restrict deep perception and exploration of living organism boundaries [31,32].

Due to the multi-scale and multi-dimensional complexity of living organism boundaries, it is difficult to accurately characterize or quantify their 4-dimensional spatiotemporal evolutionary process using single-disciplinary technical means or methods [33,34]. Therefore, there is an urgent need to search for dominant control factors, mesoscale structures, and functional parameters that originate from multiple organisms and express boundary complexity, thus discovering the common principles of living organism boundary diversity and the system competitive coordination in evolution and development. Based on this, generic mathematical models are also established to articulate or quantify the boundaries between individual biological units and mesoscale relationships.

In conclusion, the scientific community is faced with an urgent need to transcend the boundaries of disciplines and perceptions to tackle the current challenges in the development of Boundarics in Biomedicine. This necessitates the collaboration of scientists from the fields of bioscience, chemistry, medicine, materials science, and information science in living organism boundary research, without disciplinary boundaries. By uncovering new mechanisms of living organism boundary formation and evolution from an interdisciplinary perspective, we can drive the development of novel techniques and methods for cross-scale, high-sensitivity, ultra-resolution boundary imaging. This, in turn, will lead to a cutting-edge and cross-disciplinary research paradigm and field of Boundarics in Biomedicine.
